# Supplementary material for: Targeting ADAR stability: a novel approach to combat glioblastoma
Source: Front Oncol. 2026 Aug 6;16:1827793. doi: 10.3389/fonc.2026.1827793 (PMC13489870; doi:10.3389/fonc.2026.1827793)
Supplement: Supplementary file 1 [file SupplementaryFile1.zip › Supplementary file 1/Steroid_hormone_biosynthesis.pdf]

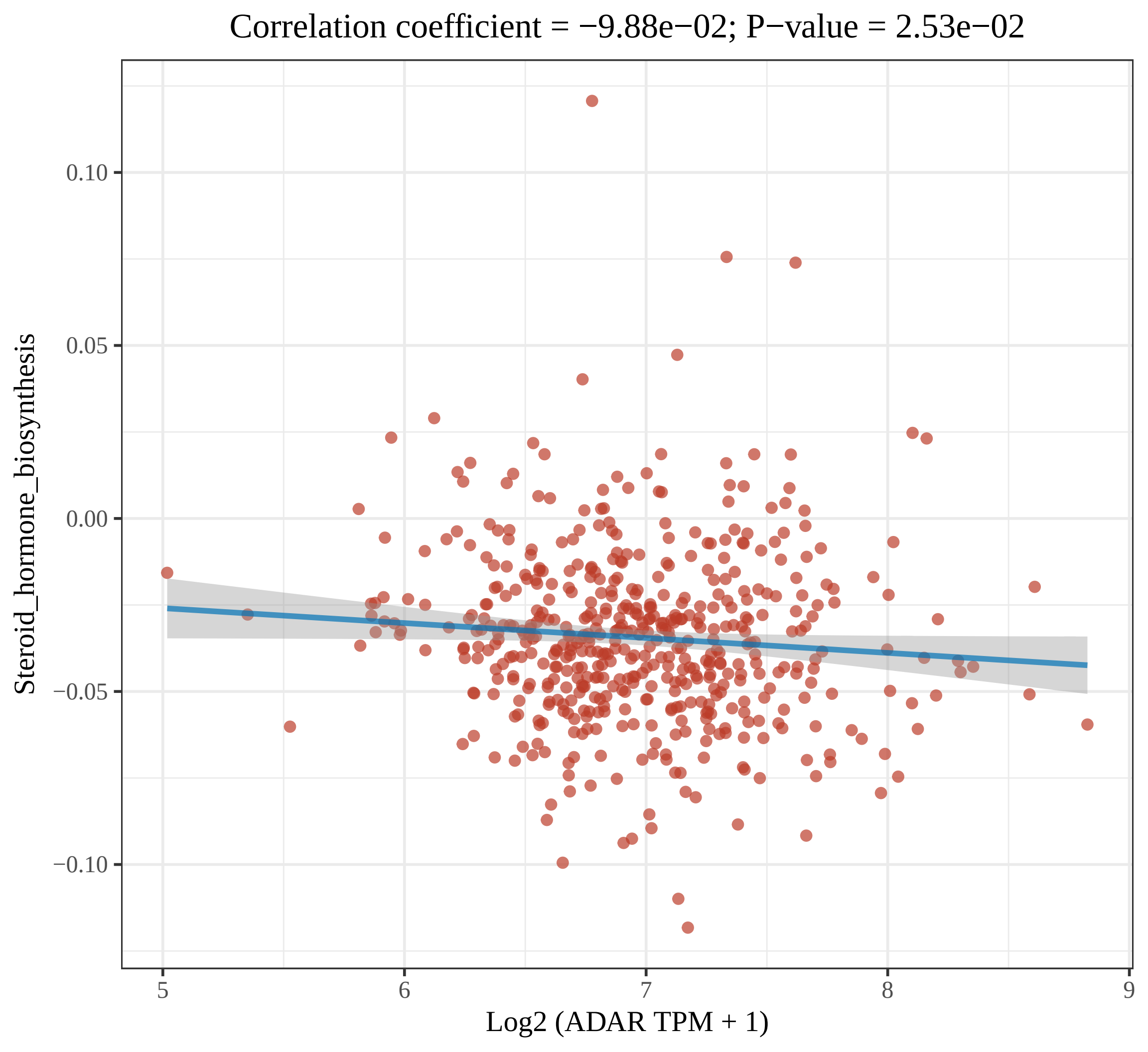
Correlation coefficient = −9.88e−02; P−value = 2.53e−02
Steroid_hormone_biosynthesis
0.10
0.05
0.00
−0.05
−0.10
5
6
7
8
9
Log2 (ADAR TPM + 1)
